# Supplementary material for: To what extent does confounding explain the association between breastfeeding duration and cognitive development up to age 14? Findings from the UK Millennium Cohort Study
Source: PLoS One. 2022 May 25;17(5):e0267326. doi: 10.1371/journal.pone.0267326 (PMC9132301; doi:10.1371/journal.pone.0267326)
Supplement: S2 Fig — (PDF) [file pone.0267326.s002.pdf]

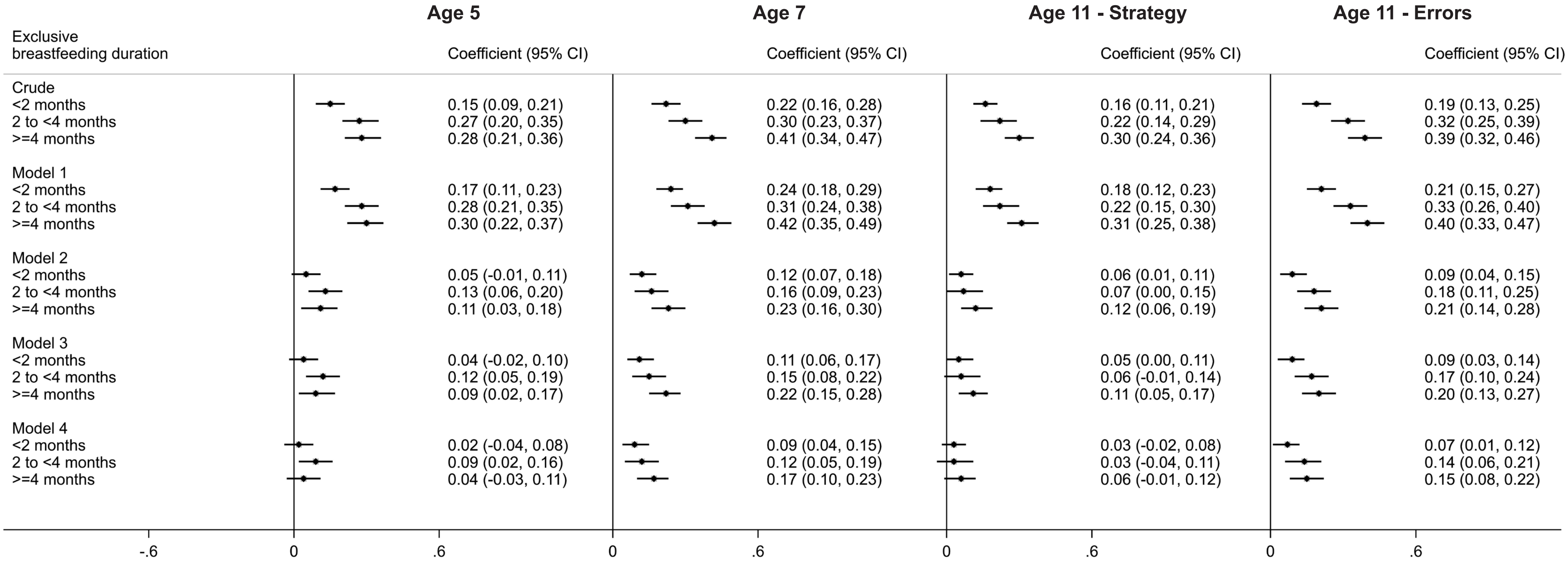

S5 Fig. Association between exclusive breastfeeding duration* and standardised cognitive spatial scores (mean: 0; SD: 1) between ages 5 and 11, UK Millennium Cohort Study (n=7,068).

*Exclusive breastfeeding (EBF) duration was defined as the time in which the child was fed with breast milk only and was based on breastfeeding duration and timing of introduction of other liquids/solids. It was classified as: Never BF; <2 months; ≥2 and <4 months, ≥4 months. All categories of EBF duration are compared to "Never breastfed" as the reference category.

Model 1: Adjusted for gestational age at birth, maternal ethnicity and languages spoken in household.
Model 2: Adjusted for Model 1 + Socioeconomic position (maternal education and highest social class in household).
Model 3: Adjusted for Model 2 + other confounding factors (older siblings in household, mother working outside the home, partnership status, maternal alcohol use during pregnancy and smoking during pregnancy).
Model 4: Adjusted for Model 3 + Maternal cognitive score.
