# Supplementary material for: To what extent does confounding explain the association between breastfeeding duration and cognitive development up to age 14? Findings from the UK Millennium Cohort Study
Source: PLoS One. 2022 May 25;17(5):e0267326. doi: 10.1371/journal.pone.0267326 (PMC9132301; doi:10.1371/journal.pone.0267326)
Supplement: S1 Table — (DOCX) [file pone.0267326.s003.docx]

**S1 Table.** Verbal cognitive scores according to the characteristics of the study subjects, UK Millennium Cohort Study (n=7,855).

| **Characteristics** | **BAS Vocabulary Age 5** | |  | **BAS Word Read. Age 7** | |  | **BAS Verbal Simil.**  **Age 11** | |  | **Word Activity Age 14** | |  |
| --- | --- | --- | --- | --- | --- | --- | --- | --- | --- | --- | --- | --- |
|  | **mean** | **(SD)** | ***P**** | **mean** | **(SD)** | ***P* *** | **mean** | **(SD)** | ***P* *** | **mean** | **(SD)** | ***P* *** |
| **Pregnancy and child-related** |  |  |  |  |  |  |  |  |  |  |  |  |
| Gestational age at birth (wk) | 0.02** | | 0.120 | 0.03** | | 0.077 | 0.00** | | 0.717 | 0.04** | | 0.003 |
| Sex |  |  |  |  |  |  |  |  |  |  |  |  |
| *Male* | -0.05 | (1.02) | 0.983 | -0.02 | (1.03) | 0.926 | -0.06 | (1.05) | 0.409 | -0.04 | (1.02) | 0.404 |
| *Female* | -0.05 | (1.05) |  | -0.03 | (1.04) |  | -0.09 | (1.02) |  | -0.06 | (1.03) |  |
| Age at cognitive test (in months, at age 5)^§^ | 0.03** | | 0.045 | -0.02** | | 0.104 | 0.00** | | 0.795 | 0.01** | | 0.327 |
| Older siblings in household |  |  |  |  |  |  |  |  |  |  |  |  |
| *Yes* | 0.06 | (1.02) | <0.001 | 0.02 | (1.00) | 0.005 | -0.03 | (1.03) | 0.004 | -0.03 | (1.04) | 0.195 |
| *No* | -0.16 | (1.04) |  | -0.07 | (1.06) |  | -0.12 | (1.04) |  | -0.07 | (1.01) |  |
| Smoked during pregnancy |  |  |  |  |  |  |  |  |  |  |  |  |
| *Never* | 0.02 | (1.09) | <0.001 | 0.09 | (1.04) | <0.001 | 0.00 | (1.04) | <0.001 | 0.03 | (1.09) | <0.001 |
| *Gave up* | 0.01 | (0.92) |  | 0.01 | (0.94) |  | -0.04 | (0.92) |  | -0.07 | (0.94) |  |
| *Kept smoking* | -0.26 | (0.94) |  | -0.37 | (0.96) |  | -0.32 | (1.03) |  | -0.27 | (0.87) |  |
| Mod/heavy alcohol in preg. |  |  |  |  |  |  |  |  |  |  |  |  |
| *Yes* | 0.01 | (1.02) | 0.265 | 0.00 | (1.03) | 0.670 | -0.08 | (1.02) | 0.897 | 0.01 | (1.06) | 0.264 |
| *No* | -0.05 | (1.04) |  | -0.03 | (1.03) |  | -0.07 | (1.03) |  | -0.05 | (1.03) |  |
| **Sociodemographic** |  |  |  |  |  |  |  |  |  |  |  |  |
| Maternal age | 0.15** | | <0.001 | 0.16** | | <0.001 | 0.14** | | <0.001 | 0.18** | | <0.001 |
| Maternal education |  |  |  |  |  |  |  |  |  |  |  |  |
| *Higher (NVQ4+5)* | 0.37 | (1.06) | <0.001 | 0.37 | (1.03) | <0.001 | 0.26 | (1.02) | <0.001 | 0.35 | (1.24) | <0.001 |
| *Medium (NVQ3)* | 0.09 | (1.03) |  | 0.14 | (0.98) |  | 0.06 | (1.00) |  | 0.08 | (1.08) |  |
| *Lower (NVQ1+2)* | -0.11 | (0.93) |  | -0.14 | (0.96) |  | -0.19 | (0.97) |  | -0.19 | (0.88) |  |
| *Other* | -0.48 | (1.20) |  | 0.05 | (1.15) |  | -0.04 | (1.12) |  | -0.11 | (1.13) |  |
| *None* | -0.60 | (0.93) |  | -0.45 | (0.94) |  | -0.43 | (0.99) |  | -0.38 | (0.80) |  |
| Highest social class |  |  |  |  |  |  |  |  |  |  |  |  |
| *Managerial/Professional* | 0.26 | (0.99) | <0.001 | 0.28 | (1.00) | <0.001 | 0.16 | (0.98) | <0.001 | 0.21 | (1.16) | <0.001 |
| *Intermediate* | -0.13 | (1.01) |  | -0.16 | (0.95) |  | -0.17 | (1.01) |  | -0.18 | (0.89) |  |
| *Semi-routine/Routine* | -0.40 | (0.92) |  | -0.40 | (0.98) |  | -0.41 | (1.02) |  | -0.34 | (0.84) |  |
| *Not applicable* | -0.57 | (1.04) |  | -0.28 | (0.97) |  | -0.26 | (0.93) |  | -0.27 | (0.85) |  |

**S1 Table (cont.).** Verbal cognitive scores according to the characteristics of the study subjects, UK Millennium Cohort Study (n=7,855).

| **Characteristics** | **BAS Vocabulary Age 5** | |  | **BAS Word Read. Age 7** | |  | **BAS Verbal Simil.**  **Age 11** | |  | **Word Activity**  **Age 14** | |  |
| --- | --- | --- | --- | --- | --- | --- | --- | --- | --- | --- | --- | --- |
|  | **mean** | **(SD)** | ***P* *** | **mean** | **(SD)** | ***P* *** | **mean** | **(SD)** | ***P* *** | **mean** | **(SD)** | ***P* *** |
| **Sociodemographic** |  |  |  |  |  |  |  |  |  |  |  |  |
| Mother working at age 9 m |  |  |  |  |  |  |  |  |  |  |  |  |
| *Yes* | 0.14 | (1.00) | <0.001 | 0.11 | (1.02) | <0.001 | 0.04 | (1.02) | <0.001 | 0.02 | (1.06) | <0.001 |
| *No* | -0.24 | (1.04) |  | -0.16 | (1.02) |  | -0.19 | (1.04) |  | -0.12 | (0.99) |  |
| Maternal partnership status |  |  |  |  |  |  |  |  |  |  |  |  |
| *Married* | 0.07 | (1.10) | <0.001 | 0.10 | (1.05) | <0.001 | 0.22 | (1.09) | <0.001 | 0.07 | (1.11) | <0.001 |
| *Cohabitation* | -0.09 | (0.90) |  | -0.11 | (0.96) |  | -0.15 | (0.93) |  | -0.13 | (0.92) |  |
| *Single mother* | -0.40 | (0.96) |  | -0.33 | (0.97) |  | -0.32 | (0.94) |  | -0.36 | (0.81) |  |
| Maternal ethnicity: White |  |  |  |  |  |  |  |  |  |  |  |  |
| *Yes* | 0.02 | (0.98) | <0.001 | -0.05 | (1.02) | 0.001 | -0.08 | (1.02) | <0.001 | -0.05 | (1.01) | 0.924 |
| *No* | -0.69 | (1.34) |  | 0.16 | (1.10) |  | 0.01 | (1.19) |  | -0.04 | (1.11) |  |
| Language spoken at home |  |  |  |  |  |  |  |  |  |  |  |  |
| *English only* | 0.01 | (0.99) | <0.001 | -0.04 | (1.02) | 0.001 | -0.07 | (1.02) | 0.866 | -0.04 | (1.01) | 0.367 |
| *English + other language* | -0.84 | (1.39) |  | 0.18 | (1.22) |  | -0.09 | (1.31) |  | -0.09 | (1.19) |  |
| **Maternal cognitive score** | 0.38** | | <0.001 | 0.31** | | <0.001 | 0.26** | | <0.001 | 0.34** | | <0.001 |

BAS: British Ability Scales.

*F test.

**Correlation

These estimates consider the complex sampling design.
