# Supplementary material for: To what extent does confounding explain the association between breastfeeding duration and cognitive development up to age 14? Findings from the UK Millennium Cohort Study
Source: PLoS One. 2022 May 25;17(5):e0267326. doi: 10.1371/journal.pone.0267326 (PMC9132301; doi:10.1371/journal.pone.0267326)
Supplement: S3 Table — (DOCX) [file pone.0267326.s005.docx]

**S3 Table.** Calculation of E-values^a^: Association between breastfeeding duration (any breastfeeding) and cognitive development, UK Millennium Cohort Study.

|  | **Original (coefficient)^b^** | **Transformed into RR scale^c^** | **E-value^d^** | **E-value, linear scale^e^** |  | **Original (coefficient) ^b^** | **Transformed into RR scale^c^** | **E-value**^d^ | **E-value, linear scale^e^** |  | **Original (coefficient) ^b^** | **Transformed into RR scale^c^** | **E-value^d^** | **E-value, linear scale^e^** |
| --- | --- | --- | --- | --- | --- | --- | --- | --- | --- | --- | --- | --- | --- | --- |
| **Breastfeeding duration** | **Verbal scores - Age 14** | | | |  | **Spatial scores - Age 11, Strategy** | | | |  | **Spatial scores - Age 11, Errors** | | | |
| **<2 months vs Never BF** | |  |  |  |  |  |  |  |  |  |  |  |  |  |
| Point estimate | -0.03 | NA | NA | NA |  | 0.00 | NA | NA | NA |  | 0.04 | NA | NA | NA |
| LL 95% CI | -0.08 | NA | NA | NA |  | -0.06 | NA | NA | NA |  | -0.02 | NA | NA | NA |
| **2-4 months vs Never BF** | |  |  |  |  |  |  |  |  |  |  |  |  |  |
| Point estimate | -0.01 | NA | NA | NA |  | 0.03 | NA | NA | NA |  | 0.11 | 1.11 | 1.45 | 0.41 |
| LL 95% CI | -0.09 | NA | NA | NA |  | -0.05 | NA | NA | NA |  | 0.03 | 1.03 | 1.20 | 0.20 |
| **4-6 months vs Never BF** | |  |  |  |  |  |  |  |  |  |  |  |  |  |
| Point estimate | 0.05 | NA | NA | NA |  | 0.10 | 1.10 | 1.43 | 0.39 |  | 0.19 | 1.19 | 1.66 | 0.56 |
| LL 95% CI | -0.03 | NA | NA | NA |  | 0.00 | - | - | - |  | 0.11 | 1.11 | 1.45 | 0.41 |
| **6-12 months vs Never BF** | |  |  |  |  |  |  |  |  |  |  |  |  |  |
| Point estimate | 0.15 | 1.15 | 1.57 | 0.50 |  | 0.06 | NA | NA | NA |  | 0.14 | 1.14 | 1.53 | 0.47 |
| LL 95% CI | 0.08 | 1.08 | 1.36 | 0.34 |  | -0.01 | NA | NA | NA |  | 0.06 | 1.06 | 1.31 | 0.30 |
| **≥12 months vs Never BF** | |  |  |  |  |  |  |  |  |  |  |  |  |  |
| Point estimate | 0.26 | 1.27 | 1.85 | 0.68 |  | 0.08 | NA | NA | NA |  | 0.15 | 1.15 | 1.57 | 0.50 |
| LL 95% CI | 0.18 | 1.18 | 1.64 | 0.54 |  | -0.01 | NA | NA | NA |  | 0.07 | 1.07 | 1.33 | 0.31 |

NA: Not applicable. LL 95% CI: Lower Limit of the 95% Confidence Interval

^a^ E-values only shown for those coefficients statistically different from zero.

^b^ Coefficient of the association between any breastfeeding duration and cognitive scores after adjustment for gestational age at birth, maternal ethnicity, languages spoken in household, socioeconomic position (maternal education and highest social class in household), older siblings in household, maternal age, mother working outside the home, partnership status, maternal smoking during pregnancy, maternal psychological distress at Age 9 months, type of child care attended by Age 9 months. mother reading to child every day at Age 5 and maternal cognitive ability.

^c^ RR ≈ exp(0.91 * d), transformation based on VanderWeele TJ, Ding P. Sensitivity Analysis in Observational Research: Introducing the E-Value. Ann Intern Med. 2017;167(4):268-74.

^d^ E-value = $RR+\sqrt{RR\cdot\left( RR-1 \right)}$, based on based on VanderWeele TJ, Ding P. Sensitivity Analysis in Observational Research: Introducing the E-Value. Ann Intern Med. 2017;167(4):268-74.

^e^ E-value expressed in SD (linear scale) for comparability.
