# Supplementary material for: To what extent does confounding explain the association between breastfeeding duration and cognitive development up to age 14? Findings from the UK Millennium Cohort Study
Source: PLoS One. 2022 May 25;17(5):e0267326. doi: 10.1371/journal.pone.0267326 (PMC9132301; doi:10.1371/journal.pone.0267326)
Supplement: S4 Table — (DOCX) [file pone.0267326.s006.docx]

**S6 Table.** Association between breastfeeding duration (any breastfeeding) and standardised cognitive verbal scores (mean: 0; SD: 1) between ages 5 and 14 among children of white English-speaking mothers, UK Millennium Cohort Study (n=6,834).

|  | **Crude** | |  | **Model 1** | |  | **Model 2** | |  | **Model 3** | |  | **Model 4** | |
| --- | --- | --- | --- | --- | --- | --- | --- | --- | --- | --- | --- | --- | --- | --- |
|  | **Coef** | **95% CI** |  | **Coef** | **95% CI** |  | **Coef** | **95% CI** |  | **Coef** | **95% CI** |  | **Coef** | **95% CI** |
| **Age 5 - BAS Vocabulary** | | |  |  |  |  |  |  |  |  |  |  |  |  |
| Never | Ref. | - |  | Ref. | - |  | Ref. | - |  | Ref. | - |  | Ref. | - |
| *<2* | 0.30 | 0.23, 0.36 |  | 0.30 | 0.23, 0.36 |  | 0.15 | 0.10, 0.22 |  | 0.12 | 0.06, 0.18 |  | 0.09 | 0.03, 0.15 |
| *≥2 to <4* | 0.36 | 0.27, 0.44 |  | 0.36 | 0.27, 0.44 |  | 0.17 | 0.09, 0.25 |  | 0.13 | 0.05, 0.21 |  | 0.08 | 0.00, 0.16 |
| *≥4 to <6* | 0.48 | 0.39, 0.56 |  | 0.47 | 0.39, 0.56 |  | 0.21 | 0.13, 0.30 |  | 0.18 | 0.09, 0.26 |  | 0.10 | 0.02, 0.18 |
| *≥6 to <12* | 0.53 | 0.44, 0.62 |  | 0.53 | 0.44, 0.61 |  | 0.25 | 0.17, 0.33 |  | 0.22 | 0.14, 0.29 |  | 0.14 | 0.06, 0.22 |
| *≥12* | 0.53 | 0.44, 0.62 |  | 0.53 | 0.44, 0.62 |  | 0.25 | 0.16, 0.33 |  | 0.21 | 0.13, 0.30 |  | 0.11 | 0.03, 0.19 |
| **Age 7 - BAS Word Reading** | | |  |  |  |  |  |  |  |  |  |  |  |  |
| Never | Ref. | - |  | Ref. | - |  | Ref. | - |  | Ref. | - |  | Ref. | - |
| *<2* | 0.27 | 0.21, 0.33 |  | 0.27 | 0.21, 0.33 |  | 0.13 | 0.07, 0.19 |  | 0.09 | 0.04, 0.15 |  | 0.06 | 0.00, 0.12 |
| *≥2 to <4* | 0.37 | 0.29, 0.45 |  | 0.37 | 0.29, 0.45 |  | 0.18 | 0.10, 0.26 |  | 0.14 | 0.06, 0.22 |  | 0.10 | 0.02, 0.17 |
| *≥4 to <6* | 0.53 | 0.44, 0.63 |  | 0.53 | 0.44, 0.63 |  | 0.27 | 0.18, 0.36 |  | 0.23 | 0.15, 0.32 |  | 0.15 | 0.07, 0.24 |
| *≥6 to <12* | 0.50 | 0.42, 0.57 |  | 0.50 | 0.42, 0.57 |  | 0.22 | 0.15, 0.28 |  | 0.18 | 0.12, 0.25 |  | 0.11 | 0.04, 0.18 |
| *≥12* | 0.55 | 0.46, 0.63 |  | 0.54 | 0.46, 0.63 |  | 0.26 | 0.18, 0.34 |  | 0.23 | 0.15, 0.31 |  | 0.12 | 0.05, 0.20 |
| **Age 11 - BAS Verbal similarities** | | |  |  |  |  |  |  |  |  |  |  |  |  |
| Never | Ref. | - |  | Ref. | - |  | Ref. | - |  | Ref. | - |  | Ref. | - |
| *<2* | 0.22 | 0.15, 0.29 |  | 0.22 | 0.15, 0.28 |  | 0.08 | 0.01, 0.14 |  | 0.04 | -0.03, 0.11 |  | 0.01 | -0.06, 0.07 |
| *≥2 to <4* | 0.26 | 0.18, 0.35 |  | 0.26 | 0.18, 0.35 |  | 0.07 | -0.01, 0.16 |  | 0.03 | -0.05, 0.12 |  | -0.01 | -0.09, 0.08 |
| *≥4 to <6* | 0.39 | 0.29, 0.48 |  | 0.38 | 0.29, 0.48 |  | 0.12 | 0.03, 0.22 |  | 0.09 | 0.00, 0.18 |  | 0.01 | -0.08, 0.10 |
| *≥6 to <12* | 0.45 | 0.37, 0.54 |  | 0.45 | 0.37, 0.53 |  | 0.17 | 0.09, 0.25 |  | 0.14 | 0.06, 0.22 |  | 0.06 | -0.02, 0.14 |
| *≥12* | 0.44 | 0.35, 0.53 |  | 0.44 | 0.35, 0.53 |  | 0.16 | 0.07, 0.24 |  | 0.12 | 0.04, 0.21 |  | 0.02 | -0.07, 0.10 |

All categories of BF duration are compared to “Never breastfed” as the reference category.

Model 1: Adjusted for gestational age at birth.

Model 2: Adjusted for Model 1 + Socioeconomic position (maternal education and highest social class in household).

Model 3: Adjusted for Model 2 + other confounding factors (older siblings in household, maternal age, mother working outside the home, partnership status, and maternal smoking during pregnancy).

Model 4: Adjusted for Model 4 + Maternal cognitive score.

**S6 Table (cont.).** Association between breastfeeding duration (any breastfeeding) and standardised cognitive verbal scores (mean: 0; SD: 1) between ages 5 and 14 among children of white English-speaking mothers, UK Millennium Cohort Study (n=6,834).

|  | **Crude** | |  | **Model 1** | |  | **Model 2** | |  | **Model 3** | |  | **Model 4** | |
| --- | --- | --- | --- | --- | --- | --- | --- | --- | --- | --- | --- | --- | --- | --- |
|  | **Coef** | **95% CI** |  | **Coef** | **95% CI** |  | **Coef** | **95% CI** |  | **Coef** | **95% CI** |  | **Coef** | **95% CI** |
| **Age 14 - Word activity** | | |  |  |  |  |  |  |  |  |  |  |  |  |
| Never | Ref. | - |  | Ref. | - |  | Ref. | - |  | Ref. | - |  | Ref. | - |
| *<2* | 0.18 | 0.12, 0.23 |  | 0.18 | 0.12, 0.23 |  | 0.04 | -0.02, 0.09 |  | 0.00 | -0.05, 0.06 |  | -0.03 | -0.08, 0.02 |
| *≥2 to <4* | 0.26 | 0.17, 0.35 |  | 0.26 | 0.17, 0.35 |  | 0.07 | -0.02, 0.16 |  | 0.03 | -0.06, 0.12 |  | -0.01 | -0.10, 0.08 |
| *≥4 to <6* | 0.42 | 0.31, 0.52 |  | 0.41 | 0.31, 0.52 |  | 0.15 | 0.06, 0.25 |  | 0.12 | 0.02, 0.21 |  | 0.04 | -0.05, 0.13 |
| *≥6 to <12* | 0.52 | 0.45, 0.60 |  | 0.52 | 0.44, 0.60 |  | 0.24 | 0.17, 0.32 |  | 0.21 | 0.14, 0.29 |  | 0.14 | 0.06, 0.21 |
| *≥12* | 0.67 | 0.58, 0.77 |  | 0.67 | 0.58, 0.77 |  | 0.39 | 0.30, 0.48 |  | 0.36 | 0.27, 0.45 |  | 0.25 | 0.16, 0.33 |

All categories of BF duration are compared to “Never breastfed” as the reference category.

Model 1: Adjusted for gestational age at birth.

Model 2: Adjusted for Model 1 + Socioeconomic position (maternal education and highest social class in household).

Model 3: Adjusted for Model 2 + other confounding factors (older siblings in household, maternal age, mother working outside the home, partnership status, and maternal smoking during pregnancy).

Model 4: Adjusted for Model 4 + Maternal cognitive score.
