# Supplementary material for: To what extent does confounding explain the association between breastfeeding duration and cognitive development up to age 14? Findings from the UK Millennium Cohort Study
Source: PLoS One. 2022 May 25;17(5):e0267326. doi: 10.1371/journal.pone.0267326 (PMC9132301; doi:10.1371/journal.pone.0267326)
Supplement: S5 Table — (DOCX) [file pone.0267326.s007.docx]

**S7 Table.** Association between breastfeeding duration (any breastfeeding) and standardised cognitive spatial scores (mean: 0; SD: 1) between ages 5 and 11 among children of white English-speaking mothers, UK Millennium Cohort Study (n=6,608).

|  | **Crude** | |  | **Model 1** | |  | **Model 2** | |  | **Model 3** | |  | **Model 4** | |
| --- | --- | --- | --- | --- | --- | --- | --- | --- | --- | --- | --- | --- | --- | --- |
|  | **Coef** | **95% CI** |  | **Coef** | **95% CI** |  | **Coef** | **95% CI** |  | **Coef** | **95% CI** |  | **Coef** | **95% CI** |
| **Age 5 - BAS Pattern Construction** | | |  |  |  |  |  |  |  |  |  |  |  |  |
| Never | Ref. | - |  | Ref. | - |  | Ref. | - |  | Ref. | - |  | Ref. | - |
| *<2* | 0.17 | 0.10, 0.24 |  | 0.17 | 0.10, 0.24 |  | 0.07 | 0.01, 0.14 |  | 0.07 | 0.00, 0.14 |  | 0.05 | -0.01, 0.12 |
| *≥2 to <4* | 0.24 | 0.15, 0.33 |  | 0.23 | 0.14, 0.32 |  | 0.11 | 0.02, 0.20 |  | 0.10 | 0.01, 0.19 |  | 0.08 | -0.01, 0.16 |
| *≥4 to <6* | 0.37 | 0.28, 0.46 |  | 0.37 | 0.28, 0.45 |  | 0.19 | 0.11, 0.28 |  | 0.18 | 0.10, 0.27 |  | 0.14 | 0.05, 0.22 |
| *≥6 to <12* | 0.30 | 0.21, 0.38 |  | 0.29 | 0.21, 0.38 |  | 0.11 | 0.02, 0.19 |  | 0.09 | 0.01, 0.18 |  | 0.05 | -0.03, 0.14 |
| *≥12* | 0.23 | 0.14, 0.31 |  | 0.22 | 0.14, 0.31 |  | 0.03 | -0.05, 0.19 |  | 0.02 | -0.06, 0.10 |  | -0.04 | -0.12, 0.04 |
| **Age 7 - BAS Pattern Construction** | | |  |  |  |  |  |  |  |  |  |  |  |  |
| Never | Ref. | - |  | Ref. | - |  | Ref. | - |  | Ref. |  |  | Ref. | - |
| *<2* | 0.22 | 0.15, 0.28 |  | 0.22 | 0.15, 0.28 |  | 0.12 | 0.06, 0.19 |  | 0.12 | 0.05, 0.18 |  | 0.10 | 0.04, 0.16 |
| *≥2 to <4* | 0.26 | 0.17, 0.35 |  | 0.26 | 0.17, 0.35 |  | 0.13 | 0.04, 0.22 |  | 0.12 | 0.04, 0.21 |  | 0.10 | 0.01, 0.19 |
| *≥4 to <6* | 0.46 | 0.37, 0.54 |  | 0.45 | 0.37, 0.54 |  | 0.28 | 0.19, 0.36 |  | -0.27 | 0.18, 0.35 |  | 0.22 | 0.14, 0.31 |
| *≥6 to <12* | 0.41 | 0.33, 0.49 |  | 0.41 | 0.33, 0.48 |  | 0.22 | 0.14, 0.30 |  | 0.21 | 0.13, 0.28 |  | 0.16 | 0.08, 0.24 |
| *≥12* | 0.41 | 0.33, 0.49 |  | 0.41 | 0.33, 0.49 |  | 0.22 | 0.14, 0.30 |  | 0.21 | 0.13, 0.29 |  | 0.15 | 0.06, 0.23 |
| **Age 11 - CANTAB Strategy** | | |  |  |  |  |  |  |  |  |  |  |  |  |
| Never | Ref. | - |  | Ref. | - |  | Ref. | - |  | Ref. | - |  | Ref. | - |
| *<2* | 0.12 | 0.06, 0.18 |  | 0.12 | 0.06, 0.17 |  | 0.02 | -0.04, 0.08 |  | 0.02 | -0.04, 0.08 |  | 0.00 | -0.06, 0.06 |
| *≥2 to <4* | 0.19 | 0.11, 0.27 |  | 0.19 | 0.11, 0.27 |  | 0.07 | -0.02, 0.15 |  | 0.06 | -0.02, 0.14 |  | 0.03 | -0.05, 0.12 |
| *≥4 to <6* | 0.35 | 0.24, 0.46 |  | 0.34 | 0.2, 0.45 |  | 0.17 | 0.06, 0.28 |  | 0.16 | 0.05, 0.26 |  | 0.11 | 0.01, 0.22 |
| *≥6 to <12* | 0.31 | 0.23, 0.38 |  | 0.30 | 0.23, 0.38 |  | 0.12 | 0.04, 0.19 |  | 0.10 | 0.03, 0.18 |  | 0.06 | -0.01, 0.14 |
| *≥12* | 0.34 | 0.24, 0.44 |  | 0.34 | 0.24, 0.43 |  | 0.14 | 0.05, 0.24 |  | 0.13 | 0.03, 0.23 |  | 0.07 | -0.03, 0.17 |

All categories of BF duration are compared to “Never breastfed” as the reference category.

Model 1: Adjusted for gestational age at birth.

Model 2: Adjusted for Model 1 + Socioeconomic position (maternal education and highest social class in household).

Model 3: Adjusted for Model 2 + other confounding factors (older siblings in household, mother working outside the home, partnership status, maternal alcohol use during pregnancy and smoking during pregnancy).

Model 4: Adjusted for Model 3 + Maternal cognitive score.

**S7 Table (cont.).** Association between breastfeeding duration (any breastfeeding) and standardised cognitive spatial scores (mean: 0; SD: 1) between ages 5 and 11 among children of white English-speaking mothers, UK Millennium Cohort Study (n=6,608).

|  | **Crude** | |  | **Model 1** | |  | **Model 2** | |  | **Model 3** | |  | **Model 4** | |
| --- | --- | --- | --- | --- | --- | --- | --- | --- | --- | --- | --- | --- | --- | --- |
|  | **Coef** | **95% CI** |  | **Coef** | **95% CI** |  | **Coef** | **95% CI** |  | **Coef** | **95% CI** |  | **Coef** | **95% CI** |
| **Age 11 - CANTAB Errors** | | |  |  |  |  |  |  |  |  |  |  |  |  |
| Never | Ref. | - |  | Ref. | - |  | Ref. | - |  | Ref. | - |  | Ref. | - |
| *<2* | 0.17 | 0.11, 0.23 |  | 0.17 | 0.11, 0.23 |  | 0.08 | 0.01, 0.14 |  | 0.07 | 0.01, 0.13 |  | 0.05 | -0.01, 0.11 |
| *≥2 to <4* | 0.28 | 0.19, 0.37 |  | 0.28 | 0.19, 0.36 |  | 0.15 | 0.06, 0.24 |  | 0.14 | 0.05, 0.23 |  | 0.12 | 0.03, 0.21 |
| *≥4 to <6* | 0.43 | 0.34, 0.52 |  | 0.43 | 0.34, 0.51 |  | 0.25 | 0.16, 0.34 |  | 0.24 | 0.15, 0.33 |  | 0.19 | 0.11, 0.28 |
| *≥6 to <12* | 0.39 | 0.31, 0.47 |  | 0.39 | 0.30, 0.47 |  | 0.20 | 0.12, 0.28 |  | 0.19 | 0.10, 0.27 |  | 0.14 | 0.06, 0.23 |
| *≥12* | 0.40 | 0.31, 0.48 |  | 0.39 | 0.31, 0.48 |  | 0.20 | 0.12, 0.29 |  | 0.19 | 0.10, 0.27 |  | 0.13 | 0.04, 0.21 |

All categories of BF duration are compared to “Never breastfed” as the reference category.

Model 1: Adjusted for gestational age at birth.

Model 2: Adjusted for Model 1 + Socioeconomic position (maternal education and highest social class in household).

Model 3: Adjusted for Model 2 + other confounding factors (older siblings in household, mother working outside the home, partnership status, maternal alcohol use during pregnancy and smoking during pregnancy).

Model 4: Adjusted for Model 3 + Maternal cognitive score.
